# Evaluation of anti-inflammatory and gastric anti-ulcer activity of *Phyllanthus niruri* L. (Euphorbiaceae) leaves in experimental rats

**DOI:** 10.1186/s12906-017-1771-7

**Published:** 2017-05-16

**Authors:** Ronia Mostofa, Shanta Ahmed, Mst. Marium Begum, Md. Sohanur Rahman, Taslima Begum, Siraj Uddin Ahmed, Riazul Haque Tuhin, Munny Das, Amir Hossain, Manju Sharma, Rayhana Begum

**Affiliations:** 1 Department of Pharmacy, Primeasia University, Dhaka, 1213 Bangladesh; 2 Department of Pharmaceutical Technology, Faculty of Pharmacy, University of Dhaka, Dhaka, Bangladesh; 3 Department of Biochemistry and Molecular Biology, University of Rajshahi, Rajshahi, 6205 Bangladesh; 4 Department of Pharmaceutical Sciences, North South University, Dhaka, 1229 Bangladesh; 5 Department of Pharmacology, Faculty of Pharmacy, Hamdard University, New Delhi, 110062 India

**Keywords:** *Phyllanthus niruri*, Phytochemical, Anti-inflammatory, Anti-Ulcer

## Abstract

**Background:**

The medicinal plants signify a massive basin of potential phytoconstituents that could be valuable as a substitute to allopathic drugs or considered as an analogue in drug development. *Phyllanthus niruri* L. (Euphorbiaceae) is generally used in traditional medicine to treat ulcer and inflammation. In this project we investigated the methanolic extract of leaves of *Phyllanthus niruri* for anti-inflammatory and anti-ulcer activity.

**Methods:**

The anti-inflammatory activity of methanol extract of *Phyllanthus niruri* leaves was evaluated at the doses of 100, 200 and 400 mg/kg, p.o. while using ibuprofen (20 mg/kg, p.o) as the standard drug. The animals used were Swiss albino rats. Inflammation was induced by injecting 0.1 ml carrageenan (1% *w*/*v*) into the left hind paw. Paw tissues from the different groups were examined for inflammatory cell infiltration. On the other hand, antiulcer activity of methanolic extract of *P. niruri* leaves at the doses of 100, 200 and 400 mg/kg, p.o. were examined against ethanol-acid induced gastric mucosal injury in the Swiss albino rats - keeping omeprazole (20 mg/kg, p.o.) as reference. The rats were dissected and the stomachs were macroscopically examined to identify hemorrhagic lesions in the glandular mucosa.

**Results:**

*P. niruri* significantly (*p* < 0.01) decreased carrageenan-induced paw edema; it exhibited a reduction of 46.80%, 55.32% and 69.14% at doses of 100, 200 and 400 mg/kg, respectively. These findings were further supported by the histological study. The methanolic extract also disclosed good protective effect against ethanol-acid induced gastric mucosal injury in the rats. Administration of the extract’s doses (100, 200 and 400 mg/kg) demonstrated a significant (*p* < 0.01) reduction in the ethanol- acid induced gastric erosion in all the experimental groups when compared to the control. The methanolic extract at the higher dose (400 mg/kg) resulted in better inhibition of ethanol-acid induced gastric ulcer as compare to omeprazole (20 mg/kg). Histological studies of the gastric wall revealed that toxic control rats revealed mucosal degeneration, ulceration and migration of numerous inflammatory cells throughout the section. On the other hand, MEPN treatment groups showed significant regeneration of mucosal layer and significantly prevented the formation of hemorrhage and edema.

**Conclusions:**

The investigation suggests that methanolic extract of *P. niruri* leaf possess anti-inflammatory activity and promotes ulcer protection as ascertained by regeneration of mucosal layer and substantial prevention of the formation of hemorrhage and edema.

## Background

Currently, various steroidal and non-steroidal anti-inflammatory drugs (NSAID) are being used to treat inflammatory diseases. Gastrointestinal bleeding and ulceration are the most recurrent and formidable problems linked with NSAID [1]. Because of these side effects, researchers are in dire need to develop safer compounds. The gastric mucosal lesions caused by ethanol, were reported as by prying with the gastric defensive mechanisms [2]. While there are many products used against gastric ulcers, most of these drugs generate several adverse reactions [3]. To study the effects of drugs on the acute phase of inflammation, models were designed to induce inflammation in rat paws by injecting pro-inflammatory agents such as carrageenan, dextran, formaldehyde etc. [4]. Carrageenan-induced paw edema animal model is usually used to assess the contribution of natural products in weathering the biochemical changes associated with acute inflammation. While the carrageenan model is typically associated with activation of the cyclooxygenase pathway and is delicate to glucocorticoids and prostaglandin synthesis antagonists, the early phase of the carrageenan reaction is due to the release of serotonin and histamine [5].

Due to the mounting concentration in the alternative therapies in current years, herbal products have become popular [6, 7]. *P. niruri* L. (Euphorbiaceae), leaves extract is one such herbal drug currently undertaken in this study primarily to explore its anti-inflammatory and anti-ulcerogenic potential in animal model. *P. niruri* can be found in the tropical regions of Asia and America. The common names of the plant are stonebreaker or seed-under-leaf. *P. niruri* is a chief plant in the Ayurvedic tradition to treat stomach, genitourinary system, liver, kidney and spleen conditions. The medicinal use of the plant in disorders includes dysentery, influenza, vaginitis, tumors, diabetes, jaundice, dyspepsia etc. The various extracts of the plant also proved to act as antiviral and antibacterial agent [8–10]. Indigenous women have also used the plant for menstruation and uterus problems [11]. Many active phytochemicals such as flavonoids, alkaloids, terpenoids, lignin, polyphenols, tannins, coumarins and saponins have been recognized from various parts of *P. niruri*. Extracts of this herb have been proven to have therapeutic effects in many preclinical studies. *Phyllanthus niruri* has been reported to be an effective anti-inflammatory [12], analgesic [13], gastroprotective [14], anti-diabetic [15], hepatoproctive [16–18], anti-malarial [19, 14] and antispasmodic [20]. In Bangladesh, *P. niruri* grows all over the country. According to a previous study, the aerial part of this plant has been reported for its anti-inflammatory activity [12]. Besides, it has been stated that the leaves of *P. niruri* contain profound amount of flavonoids and polyphenolics [21] which possess significant activity against inflammation and ulcer [22, 23]. However, there were no reports on the anti-inflammatory and antiulcer effect of *P. niruri* regarding Bangladeshi species, which encouraged us to evaluate the anti-inflammatory and antiulcer activity of *P. niruri* in rats. Because of the potentials of *P. niruri* as a medicinal plant in Bangladesh, interest in this plant is justifiable to seek anti-inflammatory and antiulcer activities. In addition the effect of *P. niruri* leave extract on inflammation and gastric ulcer was also assessed histologically.

## Methods

### Plant material

The fresh leaves of *Phyllanthus niruri* L. (Euphorbiaceae) were collected in the months of January-February 2015 from Banani, Dhaka, Bangladesh. The plant was authenticated from the Bangladesh National Herbarium, where a voucher specimen was deposited (voucher no.- 41,684).

### Drugs and chemicals

Ibuprofen and omeprazole were obtained from the pharmaceutical industry ESKAYEF BANGLADESH LIMITED. Carrageenan was obtained from Sigma Aldrich Chemicals, Germany. All other chemicals were obtained from Merck (Darmstadt, Germany) and were of analytical grade.

### Extraction procedure

Fresh leaves of *P. niruri* were cleaned and dried in an oven at 45 °C. Dried sample was pulverized to a coarse powder using a grinder. About 200 g of coarse powders were soaked in 95% methanol in a conical flask (600 ml), plugged with cotton and then covered with aluminum foil for seven days with occasional stirs. After seven days the preparation was filtered and the filtrate was collected for the preparation of extract. The filtrate was reduced by rotary evaporator and kept in normal air for few days to facilitate evaporation of the remaining solvent. The residue was then weighed (26 g) and stored in a sealed container.

### Phytochemical analysis

Phytochemistry is the branch of chemistry, deals with the chemical nature of the plant or plant products (chemistry of natural products). Plants contain many chemical constituents which are therapeutically active or inactive like carbohydrates, triterpenoids, alkaloids, glycosides, tannins, flavonoids, essential oils and other similar secondary metabolites. Qualitative phytochemical analyses were done using the standard procedures [24].

### Test for carbohydrates

#### Molisch’s test

To 2 ml of extract, 2-3 drops of alpha naphthalene solution in alcohol was added and shaken for 2 min. 1 ml of concentrated sulphuric acid was added slowly from the sides of the test tube. A deep violet colour at the junction of two layers indicated the presence of carbohydrates.

### Test for saponin

#### Foam test

The methanol extract (50 mg) was diluted with distilled water and made up to 20 ml. The suspension was shaken in a graduated cylinder for 15 min. Appearance of persistent foam indicated the presence of saponins.

### Test for alkaloids

#### Dragendorff’s test

The methanol extract (6 g.) of the plant was dissolved in 10 ml of distilled water then 2 M hydrochloric acid was added until acidify, Dragendorff’s reagent (2 ml) was added and an orange red precipitate indicated the presence of alkaloids.

### Test for glycosides

#### Borntrager’s test

For the detection of glycosides, 50 mg of methanol extract was hydrolysed with concentrated hydrochloric acid for 2 h on water bath, filtered and the hydrolysate (4 ml) of filtered hydrolysate was taken in a test tube; 6 ml of chloroform was added and shaken. Chloroform layer was separated and 10% ammonia solution was added to it pink colour indicated the presence of glycosides.

### Test for sterols/terpenes

#### Hoss’s reaction

In this test, the methanol extract (20 mg) was taken in chloroform (2 ml) and concentrated sulphuric acid was poured from side of the test tube. The colour of the ring at the junction of the two layers was noted. A violet green colour indicated the presence of cholesterol, sitosterol. A red colour ring showed the presence of sterol/terpenes.

### Test for flavonoids

#### Shimoda test

To dry methanol extract (30 mg), ethanol (2 ml) was added and dropped small piece of Magnesium ribbon. The drop wise addition of conc. HCl leads to the development of colour ranging from orange to red was confirmatory for flavonoids.

### Test for phenolics and tannins

#### Ferric chloride test

The extract (20 mg) was added in 2 ml of 1% ferric chloride solution, a purple or red colour indicated the presence of phenols.

One to 2 ml of methanol extract, a few drops of 5% aqueous ferric chloride solution was added. A bluish black colour was produced which disappears on addition of few ml of dilute sulphuric acid followed by the formation of a yellowish-brown precipitate indicated the presence of tannins.

### Test for anthraquinone

#### Borntrager test

3 ml of extract, 3 ml Benzene and 5 ml 10% ammonia solution were added and thereafter shaken properly. Appearance of a pink, red or violet colour in the ammoniacal (lower) phase was taken as the presence of free anthraquinones.

### Test for coumarin glycosides

#### NaOH test

A small amount of methanol extract was placed in test tube and covered the test tube with a filter paper moistened with dilute sodium hydroxide solution. The covered test tube was placed on water bath for several minutes. Removed the paper and exposed it to ultraviolet (UV) light, the paper showed green fluorescence.

### Anti-inflammatory activity

#### Experimental animal

Female Swiss albino rats weighing 120-150 g were used in the experiment. Animals were housed in polypropylene cages in groups of six per cage and were kept in a room maintained at 25 ± 2 °C with a 12 h light-dark cycle, and were allowed to acclimatize for one week before the experiment commenced. They were given free access to standard laboratory animal feed and water ad libitum. They were fasted over night before the experimental procedures began and all surgeries were performed under isoflurane (5% in 100% oxygen) anesthesia. The procedures were conducted with efforts to minimize preventable harm to the rats. Animal care and research protocols were centered on values and guidelines sanctioned by the Guide for the Care and Use of Laboratory Animals (NIH publication No: 85-23, revised in 1985). The prior approval for conducting the experiments on rats was obtained from the Departmental Ethics Committee of Dhaka University.

### Induction of inflammation in experimental animals

The methanolic extract of *P. niruri* (MEPN) was evaluated for anti-inflammatory activity as recommended by Winter et al. [25], injecting the edematogenic agent, explicitly carrageenan on adult albino rat [26].

There were six groups containing six rats each. Ibuprofen (20 mg/kg) was used as the reference standard. The extract was administered orally in the form of a suspension with 2 to 3 drops of tween 80 at doses of 100, 200 and 400 mg/kg respectively in the treatment group. The normal healthy group received distilled water only. All the test samples were administered orally (0.5 ml) 30 min prior to injection of carrageenan (0.1 ml of 1% *w*/*v* solution in distilled water) in the sub planted region of right paw of each rat. However, the control group received no carrageenan injection. The swelling of the paws were measured by slide calipers in one hour intervals. The observations were tabulated. The percentage of inhibition of paw edema was calculated at the end of the 6th hour.

The experimental animals were divided into the following groups and received the subsequent treatments accordingly:

**Table:** 

Groups	Treatment
Group I	0.5 ml/day distilled water, p.o.; (Normal control, NC)
Group II	0.5 ml/day distilled water, p.o. + 0.1 ml of carrageenan; (Carrageenan control, CC)
Group III	20 mg/kg/day ibuprofen, p.o. + 0.1 ml of carrageenan; (IP-20)
Group IV	100 mg/kg/day methanolic extract of *P. niruri*, p.o. + 0.1 ml of carrageenan; (MEPN-100)
Group V	200 mg/kg/day methanolic extract of *P. niruri*, p.o. + 0.1 ml of carrageenan; (MEPN-200)
Group VI	400 mg/kg/day methanolic extract of *P. niruri,* p.o. + 0.1 ml of carrageenan; (MEPN-400)

The increase in paw thickness and percentage of inhibition in control/treatment were calculated using the following formula:

$$ \mathrm{Increase}\ \mathrm{in}\ \mathrm{paw}\ \mathrm{thickness}\ \mathrm{in}\ \mathrm{control}\ \mathrm{or}\ \mathrm{treatment}\ \mathrm{group}=\mathrm{PC}\ \mathrm{or}\ \mathrm{PT}=\mathrm{Pt}\hbox{--} \mathrm{Po} $$

$$ \mathrm{Percentage}\ \mathrm{of}\ \mathrm{in}\mathrm{hibition}\ \mathrm{in}\ \mathrm{paw}\ \mathrm{thickness}\ \mathrm{in}\ \mathrm{the}\ \mathrm{treatment}\ \mathrm{group}=\mathrm{PC}\hbox{--} \mathrm{PT}\times 100/\mathrm{PC} $$

Where, Pt = paw thickness at time t, Po = initial paw thickness, PC = Increase in thickness of paw of the control group and PT = Increase in thickness of paw of the treatment group [27].

### Induction of ulcer

The animals were barred from access to any nutrients for a day and were only allowed access to drinking water for two hours before the experiment commenced. During the fasting period, the rats were placed individually in separate cages to prevent coprophagy. Thirty minutes after pre-treatment with standard (omeprazole at the dose of 20 mg/kg, p.o.) and test samples (MEPN at the doses of 100, 200 and 400 mg/kg p.o.), gastric ulcers were induced with ethanol-acid in these groups of rats (25 ml per kg of 0.3 M HCl in 60% ethanol) [28]. These rats were sacrificed 90 min after induction and their stomachs were immediately excised. Each stomach was opened along the larger curvature, washed with distilled water. The gastric mucosa was examined for ulcers by magnifying lens and scoring of ulcer was made as follows [29].

**Table:** 

Scoring of Ulcer					
Normal stomach	Red coloration	Spot ulcer	Hemorrhagic streak	Ulcers	Perforation
0	0.5	1	1.5	2	3

Mean ulcer score for each animal was expressed as ulcer index. The percentage of ulcer protection was determined as follows:-

$$ \%\mathrm{protection}=\frac{\mathrm{control}\ \mathrm{mean}\ \mathrm{ulcer}\ \mathrm{index}\hbox{-} \mathrm{test}\ \mathrm{mean}\ \mathrm{ulcer}\ \mathrm{index}}{\mathrm{control}\ \mathrm{mean}\ \mathrm{ulcer}\ \mathrm{index}}\times 100 $$

The experimental animals were divided into six groups, each consisting of six rats and received following treatment:

**Table:** 

Groups	Treatment
Group 1	5 ml/kg/day distilled water, p.o.; (Normal control)
Group 2	5 ml/kg/day distilled water, p.o. + 25 ml per kg of 0.3 M HCl in 60% ethanol; (Ethanol control)
Group 3	20 mg/kg/day omeprazole, p.o. + 25 ml per kg of 0.3 M HCl in 60% ethanol;
Group 4	100 mg/kg/day methanolic extract of *P. niruri*, p.o. + 25 ml per kg of 0.3 M HCl in 60% ethanol;
Group 5	200 mg/kg/day methanolic extract of *P. niruri*, p.o. + 25 ml per kg of 0.3 M HCl in 60% ethanol;
Group 6	400 mg/kg/day methanolic extract of *P. niruri*, p.o. + 25 ml per kg of 0.3 M HCl in 60% ethanol;

### Histological investigation

At the end of the studies animals were sacrificed while they were under isoflurane (5% in 100% oxygen) anaesthesia. For histological examination, paw tissues were taken 6 h after edema was induced by carrageenan. The tissue slices were immediately fixed in freshly prepared 10% neutral buffered formalin for a minimum of 24 h. On the other hand, specimens of the gastric walls from each rat were kept in 10% buffered formalin for 24 h for histopathological examination following the assessment of ulcer score for anti-ulcer activity. Then the tissue specimens were processed for paraffin embedding tissue sections. The samples were sectioned with a microtome, stained with hematoxyline and Eosin (H and E) and mounted on Canada balsam. All sections were examined under light microscope. Photographs of the lesions were taken with an Olympus photo microscope for observation and documentation of histopathological changes such as oedema, inflammation, infiltration and erosion.

### Statistical analysis

The values are represented as mean ± S.E.M, and statistical significance between treated and control groups was analyzed using One way ANOVA, followed by Dunnett’s test where *P* < 0.05 was considered statistically significant.

## Results

### Preliminary phytochemical analysis

The traditional use of the species was scientifically validated through the identification of the phytochemicals responsible for their use in indigenous systems of health care. The result of qualitative chemical analysis of the methanolic extract of *P. niruri* is tabulated in Table 1.

**Table 1:** Preliminary phytochemical analysis of *P. niruri* leaves extract

Phytoconstituents	Methanolic extract of *P. niruri*
Carbohydrates	+
Saponins	+
Alkaloids	+
Glycosides	**–**
Terpenoids	+
Steroids	+
Flavonoids	+
Phenolics and Tannins	+
Anthraquinone	**–**
Coumarins glycosides	+

+ = Present, − = Absent

### Carrageenan induced acute inflammation

**Fig. 1:**
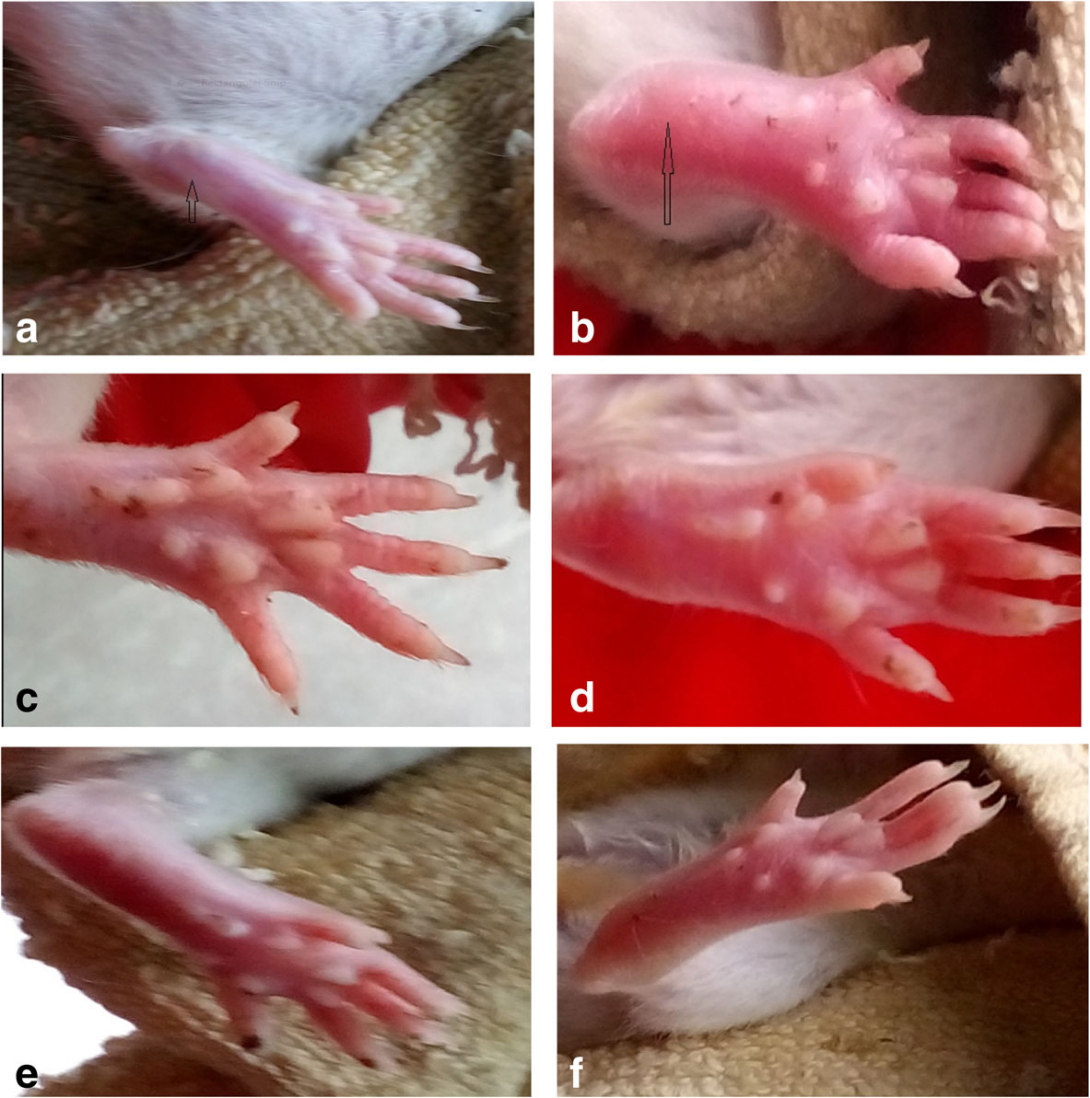
Effect of methanolic extract of *P. niruri* on carrageenan induced paw edema after 6 h. **a** = Normal control: no erythema and inflammation were observed. **b** Carrageenan control: severe erythema and swelling were observed. **c** 20 mg/kg ibuprofen: mild amount of erythema and swelling were observed. **d** 100 mg/kg methanolic extract of *P. niruri*: moderate amount of erythema and swelling were observed **e** 200 mg/kg methanolic extract of *P. niruri*: moderate amount of erythema and swelling were observed. **f** 400 mg/kg methanolic extract of *P. niruri*: mild amount of erythema and swelling were observed. The small arrow indicates the absence of swelling and erythema whereas the large arrow indicates severe swelling and erythema in the rats paw

Ibuprofen was used as the reference drug during the anti-inflammatory evaluation of the methanolic extract of the leaves of *P. niruri* in carrageenan induced acute inflammation model. Animals that were treated with ibuprofen (20 mg/kg, p.o.) and methanolic extract (100, 200 and 400 mg/kg p.o.) exhibited significant reduction in paw thickness from 1st to the 6th hour (Table 2). After 6 h of carrageenan treatment, swelling and redness were observed in carrageenan control group, while swelling and redness were significantly reduced in the groups which were given MEPN. Fig. 1 showed the images of the inflamed paw of the groups viz., carrageenan control, standard and treatment groups at 6 h after carrageenan injection. The results obtained at the end of the study disclosed that the extract exhibited significant (*p* < 0.01) anti-inflammatory activity in inflamed rat paws, when compared with carrageenan control. At the end of the study, ibuprofen (20 mg/kg) treated group showed 64.89% of inhibition. Oral administration of methanolic extract at the doses of 100, 200 and 400 mg/kg reduced paw edema by 46.80, 55.32 and 69.14%, respectively when compared with control group at the 6^th^ hour after carrageenan injection.

**Table 2:** Anti-inflammatory effect of *Phyllanthus niruri* methanolic extract on carrageenan induced changes in paw thickness in experimental rats

Groups	NC	CC	IP-20	MEPN-100	MEPN-200	MEPN-400
Dose mg/kg	---------	-------	20 mg/kg ibuprofen	100 mg/kg extract	200 mg/kg extract	400 mg/kg extract
Initial paw thickness (cm)	0.49 ± 0.07	0.42 ± 0.06	0.45 ± 0.09^b*^	0.44 ± 0.05^b*^	0.44 ± 0.08^b*^	0.43 ± 0.06^b*^
Paw thickness (cm) after 1 h	0.49 ± 0.05	0.74 ± 0.08^a*^	0.52 ± 0.07^b*^	0.6 ± 0.07^b,c^	0.58 ± 0.05^b,c^	0.51 ± 0.07^b*^
Paw thickness (cm) after 2 h	0.49 ± 0.08	0.83 ± 0.07^a**^	0.57 ± 0.05^b**^	0.67 ± 0.04^b,c^	0.63 ± 0.05^b*^	0.57 ± 0.06 ^b**^
Paw thickness (cm) after 3 h	0.49 ± 0.05	0.91 ± 0.09^a**^	0.62 ± 0.10^b**^	0.7 ± 0.06^b,c^	0.66 ± 0.06^b*^	0.6 ± 0.08^b**^
Paw thickness (cm) after 4 h	0.49 ± 0.06	0.99 ± 0.11^a**^	0.58 ± 0.10^b**^	0.68 ± 0.09^b*^	0.62 ± 0.07^b**^	0.56 ± 0.09 ^b**^
Paw thickness (cm) after 5 h	0.49 ± 0.08	0.96 ± 0.09^a**^	0.49 ± 0.07^b**^	0.65 ± 0.10^b*^	0.59 ± 0.07^b**^	0.48 ± 0.06 ^b**^
Paw thickness (cm) after 6 h	0.48 ± 0.06	0.94 ± 0.07^a**^	0.33 ± 0.09^b**^	0.50 ± 0.06^b**^	0.42 ± 0.07^b**^	0.29 ± 0.07 ^b**^

Each value is Mean ± S.E.M (*n =* 6). (*) indicates statistically significant difference from respective group using one way analysis of variance, followed by Dunnett’s multiple comparison test (*p < 0.05 and ^**^ *p* < 0.01). ^c^indicates statistically no significant difference from respective group using one way analysis of variance, followed by Dunnett’s multiple comparison test (*p* > 0.05). ^a^when compared with normal control, ^b^when compared with carrageenan control; NC- Normal control, CC- Carrageenan control, IP-20 – ibuprofen 20 mg/kg/day, MEPN-100 -methanolic extract of *P. niruri* 100 mg/kg/day, MEPN-200- methanolic extract of *P. niruri* 200 mg/kg/day, MEPN-400 - methanolic extract of *P. niruri* 400 mg/kg/day.

### Histopathology of paw tissue

**Fig. 2:**
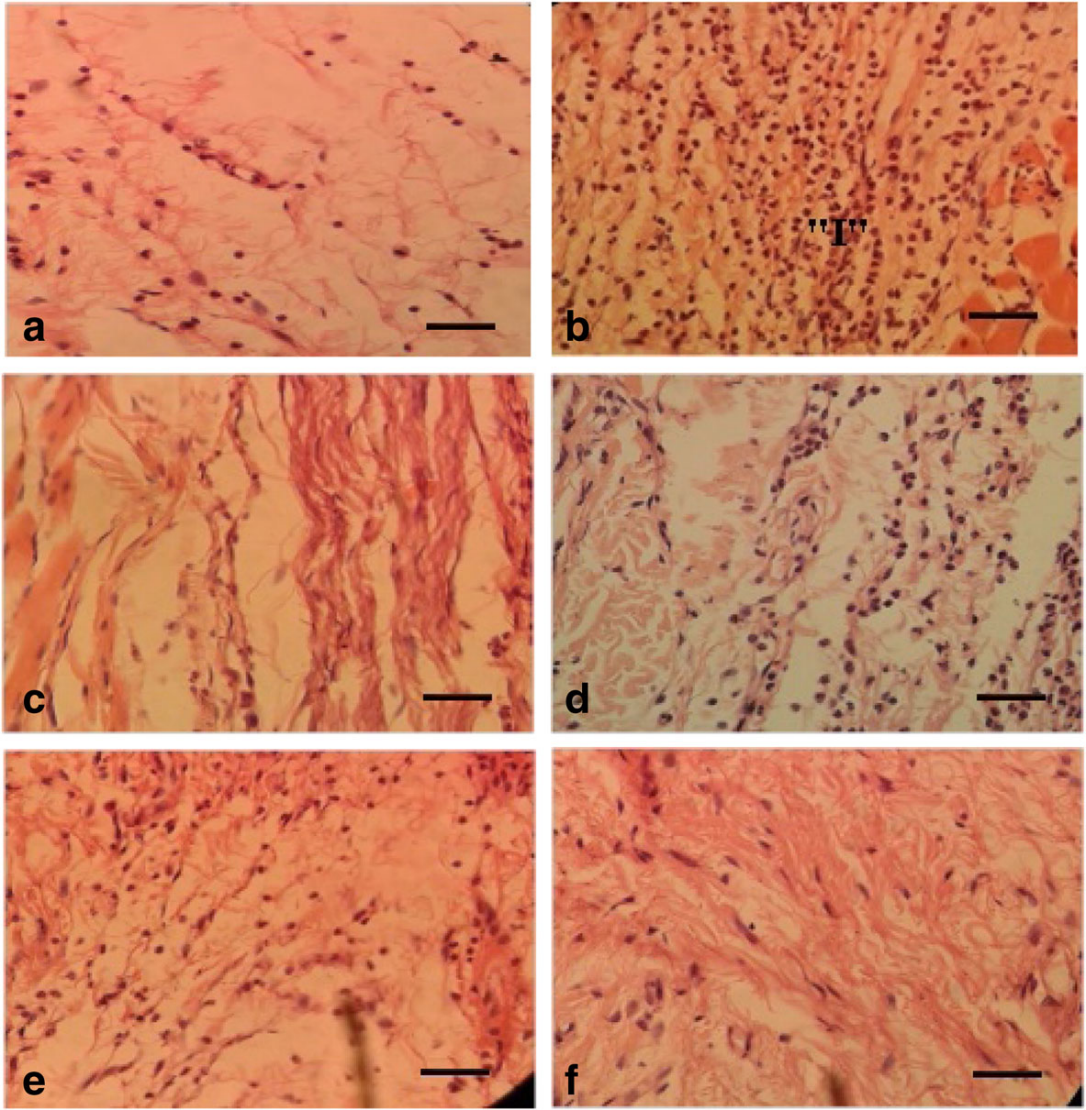
Histological evaluation of anti-inflammatory effects of methanolic extract of *P. niruri* **a** Normal control**, b** Carrageenan control, **c** 20 mg/kg ibuprofen, **d** 100 mg/kg methanolic extract of *P. niruri*, **e** 200 mg/kg methanolic extract of *P. niruri*, **f** 400 mg/kg methanolic extract of *P. niruri*. Each group was assessed at 400× magnification, scale bar: 20 μm; “I” indicates numerous infiltrations of inflammatory cells in the specimen from the rats paw tissue

Fig. 2a showed a section from the rat paw received only distilled water without carrageenan injection. The tissue architecture was preserved, showing dermal collagen and minimal number of leukocytes. Fig. 2b demonstrated the carrageenan induced section, elicit migration of numerous inflammatory cells throughout the section was observed. On the other hand, ibuprofen (20 mg/kg) treated group revealed appearance of only few inflammatory cells (Fig. 2c). However, groups treated with methanolic extract of *P. niruri* at doses of 100 and 200 mg/kg displayed moderate to minimal number of inflammatory cell infiltration (Fig. 2d, e, respectively). The inflammatory cells’ infiltration was almost completely reduced by the treatment with methanolic extract of *P. niruri* at the dose of 400 mg/kg when compared with CC group (Fig. 2f).

### Effect of *P. niruri* on ethanol-induced gastric ulcer

In ethanol control animal, oral administration of ethanol produced characteristic lesions in the glandular portion of rat’s stomach which appeared as elongated bands of thick, black & dark red lesions. MEPN showed significant protection index of 69.59, 74.32 and 80.40% with the dose of 100, 200 and 400 mg/kg/day, p.o. respectively in comparison to ethanol control. Whereas omeprazole (standard drug) reduced ulcer by 75.00% (Results are tabulated in Table 3).

**Table 3:** Effect of *P. niruri* leaves extract on various parameters in ethanol induced gastric ulcer

Groups	Ulcer index	% Protection
Normal control	1.56 ± 0.04	-
Ethanol control	14.8 ± 1.03^a**^	-
Omeprazole 20 mg/kg	3.7 ± 0.06^b**^	75.00
MEPN 100 mg/kg	4.5 ± 0.05^b**^	69.59
MEPN 200 mg/kg	3.8 ± 0.03^b**^	74.32
MEPN 400 mg/kg	2.9 ± 0.05^b**^	80.40

MEPN- methanolic extract of *P. niruri*

Each value is Mean ± S.E.M (*n =* 6). (*) indicates statistically significant alteration from respective group using one way analysis of variance followed by Dunnett’s multiple comparison test (^**^ *p* < 0.01). ^a^when compared with normal control, ^b^when compared with ethanol control

### Gross evaluations of gastric lesions

**Fig. 3:**
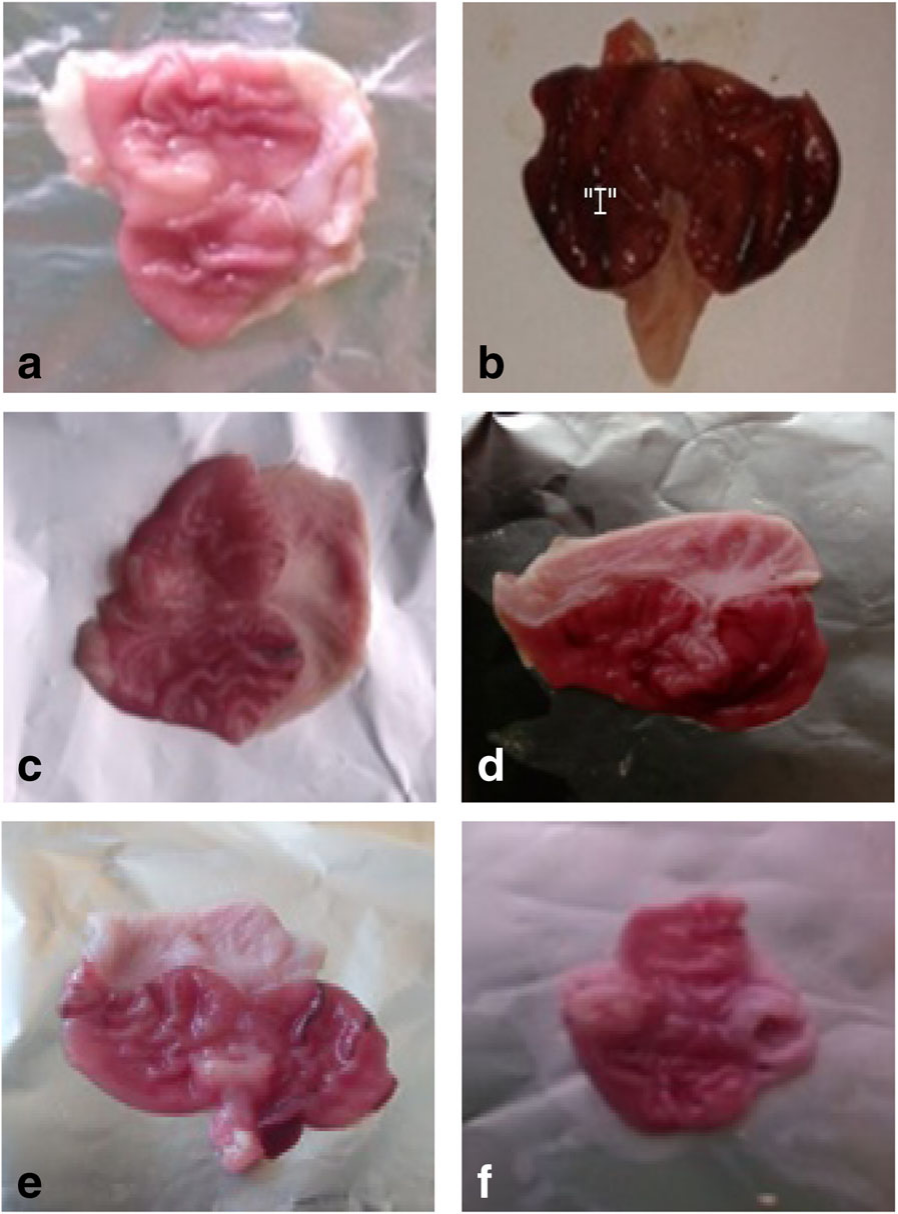
Gross appearance of the gastric mucosa. **a** Normal control: no mucosal damage was observed**, b** Ethanol control: marked ulcers along with hemorrhagic streaks and mucosal damage were observed, **c** 20 mg/kg omeprazole, mild injuries were observed in the gastric mucosa as compared to the ethanol control group, **d** 100 mg/kg methanolic extract of *P. Niruri* **:** moderately reduced gastric mucosal damage and ulcers were observed, **e** 200 mg/kg ethanolic extract of *P. Niruri*: significantly reduced gastric mucosal damage and ulcers were observed, **f** 400 mg/kg methanolic extract of *P. Niruri*: no damage to the gastric mucosa was observed and gastric mucosa appeared flat as compared to the ethanol control. “I” indicates gastric mucosal damage and hemorrhagic streaks

Ethanol controlled rats exhibited severe mucosal injury whereas, the rats that were treated with *P. niruri* leaves extract before ethanolic induction had significantly reduced areas of gastric ulceration revealing flattening of gastric mucosal folds compared to rats treated with only distilled water. There were no significant differences between doses of 200 and 400 mg/kg methanolic extract in terms of area of ulceration. It was also observed that protection of gastric mucosa was more prominent in rats treated with 400 mg/kg methanolic extract (Fig. 3).

### Histological evaluation of gastric lesions

**Fig. 4:**
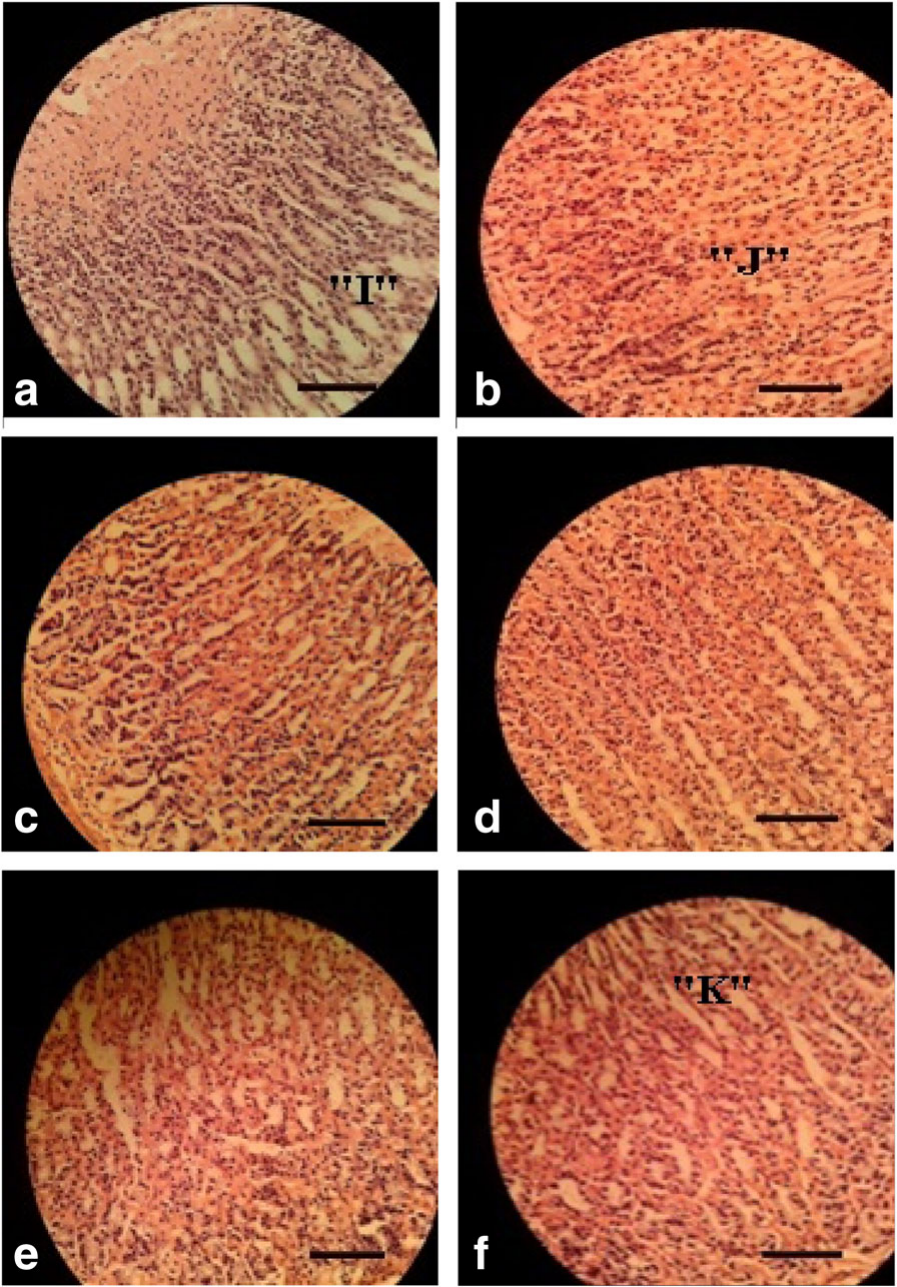
Histological evaluation of anti-ulcer effect of methanolic extract of *P. niruri.* **a** Normal control, **b** Ethanol control, **c** 20 mg/kg omeprazole, **d** 100 mg/kg methanolic extract of *P. Niruri* **, e** 200 mg/kg methanolic extract of *P. Niruri*, **f** 400 mg/kg methanolic extract of *P. Niruri*. Each group was assessed at 400× magnification, scale bar: 20 μm; “I” indicates normal gastric mucosa, “J” indicates degeneration of gastric mucosa and infiltration of inflammatory cells frequently observed in the specimen from the stomach, “K” indicates almost complete regeneration of gastric mucosa

Fig. 4a shows a section from the subject that received only distilled water without induction of ulcer. The section of gastric mucosal layer showed normal tissue architecture and absence of gastric tissue degeneration. Whereas the ethanol control group demonstrated mucosal degeneration, ulceration and migration of numerous inflammatory cells throughout the section (Fig. 4b). However, administration of omeprazole (20 mg/kg) showed no significant change in histopathology and in turn revealed regeneration of structure and prevention of hemorrhage and edema (Fig. 4c). MEPN at the dose of 100 mg/kg exhibited moderate regeneration (Fig. 4d). On the other hand, MEPN at the doses of 200 and 400 mg/kg displayed significant regeneration of mucosal layer and expressively prevented the development of hemorrhage and edema (Fig. 4e, f respectively).

## Discussion

Upon phytochemical screening the methanolic extract of *P. niruri* disclosed the presence of alkaloids, phenols, steroids, triterpinoids, flavonoids and coumarins. Many studies have reported that certain terpenoids, steroids and phenolic compounds (tannins, coumarins and flavonoids) have protective effects due to their antioxidant properties. [30–32]. Lately, a number of natural products of traditional medicines and ingredients of healthy foods have been comprehensively explored and subjected to clinical trials to establish as anti-inflammatory agents [33]. Presence of major Phytoconstituents in the methanolic extract of leaves of *P. niruri* makes it a potential candidate for further investigation.

The edema induced by carrageenan was expressed in two phases (first phase and second phase) [34]. In the first phase: a rapid rise in edema was detected instantly after sub-plantar injection of carrageenan. In the second phase (at the end of 2nd hour), a significant increase in edema was detected. The release of prostaglandins is thought to be the main reason for the swelling in second phase [35]. In this study, MEPN inhibited the carrageenan induced edema in a dose-dependent manner and had a potential anti-inflammatory effect in the second phase (2^nd^-6^th^ hour). In the treatment groups, the development of inflammation in the second phase was less. MEPN might have demonstrated their anti-inflammatory activity by inhibiting the synthesis and release of prostaglandins, proteases, and lysosomal enzymes.

In the present study, the histopathogical examination of the hind paw tissue showed that methanolic extract of *P. niruri* suppressed the massive influx and accumulation of inflammatory cells in the paw tissue after carrageenan induction. The suppressive effects were observed at all doses of the test drugs. However, the present investigation concluded that methanolic extract of *P. niruri* reduced the inflammatory cells infiltration, in a dose-dependent manner and at the higher dose the effect was similar to that of reference drug.

The anti-ulcer effect of the methanolic extract was evaluated using ethanol induced gastric ulcer model. Ethanol induced gastric lesions formed due to interference in gastric blood flow which contributes to the development of the hemorrhage and necrotic aspects of tissue injury. Alcohol swiftly penetrates the gastric mucosa superficially causing cell and plasma membrane damage leading to augmented intracellular membrane permeability to sodium and water. The mammoth buildup of calcium describes a chief step in the pathogenesis of gastric mucosal injury. This sequence leads to the demise of cells and erosion of epithelium’s surface [36, 37].

The results revealed that the ethanol administration in the control group resulted in immense ulceration in comparison with the normal group. However, treatment with omeprazole at the dose of 20 mg/kg and methanolic extract of *P. niruri* at the doses of 100,200 and 400 mg/kg prior to ethanol administration exhibited significant inhibition. Among the test samples, the best result was obtained with *P. niruri* at an optimum dose of 400 mg/kg which was potentially effective as compared to the standard drug, omeprazole. Edema, cellular debris and damaged mucosal epithelium were found in ulcerated stomach membranes. Protections against these histopathological changes by MEPN in pre-treated rats were observed, similar to the result of omeprazole.

However, the findings observed in the current studies support and extend previous results that reported the anti-inflammatory and anti-ulcer activities of *Phyllanthus niruri* aerial part and leave extract, respectively. Furthermore, the present studies also revealed a better inhibition of inflammation and gastric ulcer as compare to the previously reported.

## Conclusion

In our study the extract exhibited protection against characteristic lesions produced by ethanol administration. This antiulcer effect of methanolic extract of *P. niruri* may be due to both reductions in gastric acid secretion and gastric cytoprotection. Further studies are needed for their exact mechanism of action on gastric acid secretion and gastric cytoprotection. However, the present investigation concluded that the treatment of extracts reduced the ethanol induced ulcer in a dose-dependent manner and at the higher dose (400 mg/kg) the effect was similar to that of reference drug.

In conclusion, MEPN exhibited anti-inflammatory and antiulcerogenic activity. MEPN at the dose of 400 mg/kg showed higher level of cytoprotection. The depletion in inflammation may have occurred due to high flavonoid, triterpenoids, steroids, saponins and tannin content. However, the mechanisms behind these events are still vague. Therefore, further experiments should be undertaken to identify which of the phytoconstituents and mechanisms are involved in the actions illustrated by the results.

## Abbreviations

*P*: *Phyllanthus*
MEPN: methanolic extract of leaves of *Phyllanthus niruri*
NC: Normal control
CC: Carrageenan control
NSAID: non-steroidal anti-inflammatory drugs
